# Supplementary material for: Collaborative intelligence in AI: Evaluating the performance of a council of AIs on the USMLE
Source: PLOS Digit Health. 2025 Oct 9;4(10):e0000787. doi: 10.1371/journal.pdig.0000787 (PMC12510544; doi:10.1371/journal.pdig.0000787)
Supplement: S2 File — (ZIP) [file pdig.0000787.s002.zip › S2 Data Raw data files containing unprocessed question inputs and LLM outputs including transcripts of deliberations/STEP3_Question-58-1_Type-MC-NJ.docx]

**Facilitator** **[Round 1]:** A case-control study is conducted to assess risk factors predicting inpatient mortality among geriatric patients with community-acquired pneumonia. Results of the study include the odds ratios shown below, which were calculated from a multivariable logistic regression equation: Predictor Variables: Hypotension (systolic blood pressure ≤100 mm Hg) - Odds Ratio: 3.32, 95% CI (1.65 to 5.11) Hypoxemia (PO2 ≤ 50 mm Hg) - Odds Ratio: 2.43, 95% CI (1.55 to 3.32) Lung infiltrate present on chest x-ray at time of admission - Odds Ratio: 1.35, 95% CI (0.76 to 2.24) When the other covariates are controlled, which of the following is the most appropriate conclusion regarding these data? (A) The risk for inpatient mortality is greater for patients with hypotension than for those without hypotension (B) The risk for inpatient mortality is increased more by hypoxemia than by hypotension (C) The risk for inpatient mortality is increased when there is a pulmonary infiltrate present on chest x-ray at the time of admission (D) The risk for inpatient mortality is significantly affected by all of these predictor variables

**AI_B:** RESTATING THE QUESTION: The presented data is from a case-control study, which evaluated certain risk factors to predict inpatient mortality among older individuals with community-acquired pneumonia. The predictor variables being analyzed were presence of hypotension (defined as systolic blood pressure ≤100 mm Hg), hypoxemia (PO2 ≤ 50 mm Hg), and lung infiltrates visible on chest x-ray upon admission. The odds ratios for these variables were calculated using a multivariable logistic regression analysis with the inclusion of other unnamed covariates. PLANNING: The first assumption is that all of these predictor variables significantly increase mortality risk in these patients. This can be questioned by examining the odds ratios and confidence intervals given in the results. For instance, a predictor variable would only significantly increase the risk of inpatient mortality if its odds ratio was significantly above 1, as this indicates a direct correlation with the predicted outcome. Additionally, the confidence interval should not include 1 for the factor to be considered significant. CLINICAL REASONING: It's worth noting that the odds ratio for hypotension is highest at 3.32 and its 95% confidence interval doesn't include 1, which suggests that this factor significantly increases inpatient mortality. However, while hypoxemia also has an odds ratio above 1 (2.43) and its 95% confidence interval does not include 1, its odds ratio is lower than that of hypotension, suggesting it is less impactful on mortality. For lung infiltrates visible at the time of admission, the odds ratio is 1.35, but its 95% CI dips below 1, which means it may not significantly affect inpatient mortality risk. CONCLUDING: After carefully analyzing the provided data, it can be concluded that both hypotension and hypoxemia significantly increase risk and could be associated with higher inpatient mortality among older patients with community-acquired pneumonia, with hypotension having a greater influence. However, the presence of lung infiltrates detected via a chest x-ray at the time of admission may not be a significant predictor of inpatient mortality, as suggested by its confidence interval. RESPONSE: Hence, given the information above, the most appropriate conclusion would be: (A) The risk for inpatient mortality is greater for patients with hypotension than for those without hypotension.

**AI_D:** RESTATING THE QUESTION: A case-control study was performed to evaluate risk factors for in-hospital death in older individuals who were suffering from pneumonia that was contracted within the community. The study identified three predictor variables for inpatient mortality, namely hypotension, hypoxemia, and the presence of a lung infiltrate upon admission. An odds ratio (OR) was calculated for each variable. The OR is a statistic that quantifies the strength of the association between a predictor variable and an outcome. A higher OR indicates a stronger association. The question wants to interpret these odds ratios when all other variables are kept constant, also known as covariates. PLANNING: It is important to look at the odds ratios (ORs) and their 95% confidence intervals (CIs) to evaluate the conclusions. If the OR is greater than 1, and the lower bound of the 95% CI is also above 1, then it is assumed that the predictor variable significantly increases the risk of the outcome. This means that individuals with the predictor variable are at a higher risk of experiencing the outcome than those without it. Conversely, if the lower bound of the 95% CI includes or is below 1, the predictor variable does not significantly increase the risk for the outcome. CLINICAL REASONING: Looking at the ORs and their corresponding 95% CIs for the predictor variables: 1) Hypotension - OR: 3.32, 95% CI (1.65 to 5.11): This indicates individuals with hypotension have more than three times the odds of inpatient death compared to those without hypotension. And as the 95% CI is above 1, this is a significant increase. 2) Hypoxemia - OR: 2.43, 95% CI (1.55 to 3.32): Similar to hypotension, hypoxemic patients also face an increased risk of inpatient mortality. Despite the OR being lower than that for hypotension, the 95% CI still does not include 1, indicating a significant increase. 3) Lung infiltrate - OR: 1.35, 95% CI (0.76 to 2.24): Here, the lower bound falls under 1, which means this predictor variable does not statistically significantly increase the risk for inpatient death. CONCLUDING: Upon analyzing the data, hypotension and hypoxemia show statistically significant increases in inpatient mortality risk. However, the presence of a lung infiltrate at the time of admission does not essentially increase the risk since the lower bound of the confidence interval includes 1. RESPONSE: Therefore, the most appropriate conclusion is (A) The risk for inpatient mortality is greater for patients with hypotension than for those without hypotension.

**AI_E:** RESTATING THE QUESTION: This case-control study aims to determine the risk factors that predict inpatient mortality in older patients with pneumonia contracted within their communities. The predictor variables include hypotension, hypoxemia, and lung infiltrate, with each one having different odds ratios, which represents the odds of dying in-hospital for those with the condition versus those without. The query here seeks to identify the most valid conclusion that can be drawn from these data when other factors are held constant. PLANNING: Let's examine the assumptions. First, regarding hypotension, the odds ratio of 3.32 suggests patients with hypotension have a higher risk of in-hospital mortality than those without hypotension. Second, for hypoxemia, with an odds ratio of 2.43, we may assume that the risk for mortality is increased in the presence of hypoxemia. Third, when lung infiltrates are present on chest x-ray at admission, with an odds ratio of 1.35, it could be deduced that these infiltrates also elevate the inpatient mortality risk. Finally, we could assume that all the mentioned predictor variables significantly affect the risk for mortality. However, we have to consider the 95% confidence intervals (CI) of the estimates, which provide a plausible range of the true odds ratios. An interval that crosses 1 indicates that the predictor may not have a significant effect. CLINICAL REASONING: The critical factor in the interpretation of the odds ratios is the 95% confidence interval. If the interval includes 1, there's a possibility that the variable has no significant association with the outcome, because the odds ratio of 1 represents a null effect i.e., the event is just as likely in one group as in the other. In our case, the odds ratios for both hypotension (3.32; 1.65 to 5.11) and hypoxemia (2.43; 1.55 to 3.32) are significantly above 1, and their CIs do not include 1, indicating a significant association with inpatient mortality. However, for the presence of lung infiltrate on chest x-ray at time of admission (1.35; 0.76 to 2.24), the CI includes 1, indicating that this variable may not significantly affect mortality. CONCLUDING: Summarizing the findings from the case-control study and the interpretation of the odds ratios, it can be concluded that hypotension and hypoxemia significantly increase the risk of inpatient mortality in geriatric patients with community-acquired pneumonia. Whereas, the presence of lung infiltrates on chest x-ray at the time of admission does not seem to have a significant association with inpatient mortality. RESPONSE: Therefore, the answer is (A) The risk for inpatient mortality is greater for patients with hypotension than for those without hypotension. The odds ratio suggests that hypotension presents a higher risk factor compared to those without hypotension, while the presence or absence of pulmonary infiltrate does not significantly alter the mortality risk in these patients.

**AI_C:** RESTATING THE QUESTION: The case-control study aims to understand which factors increase the likelihood of death in hospitalized elderly patients suffering from community-acquired pneumonia. Three predictors were analyzed, hypotension, hypoxemia, and the presence of lung infiltrates, using a multivariable logistic regression equation. An odds ratio was established for each predictor. The lower the threshold of blood pressure and oxygenation (PO2) denotes hypotension and hypoxemia, respectively. Lung infiltrates refer to opacities or whitening seen on a chest x-ray, often a manifestation of pneumonia. The higher the odds ratio, the stronger the association of that factor with increased mortality risk. The question wants to deduce the most accurate interpretation of the data given the odds ratios and their respective 95% confidence intervals. PLANNING: The assumptions here are that the variables with the higher odds ratios would significantly affect the risk of inpatient mortality, and the predictor factors with odds ratios greater than 1 would increase the mortality risk. However, several factors such as the width of the confidence interval and whether it includes 1 should also be considered. In evaluating these assumptions, it is important to note that the odds ratio of 1 implies no association between exposure and outcome. A wide confidence interval indicates more data variability. CLINICAL REASONING: Looking closer at the data, hypotension (3.32, cover 1.65–5.11) and hypoxemia (2.43, cover 1.55–3.32) have a significant association with mortality as their odds ratios are greater than 1, and their 95% confidence intervals do not cross1. The higher value of the odds ratio for hypotension suggests a stronger association with mortality when compared to hypoxemia. On the other hand, pulmonary infiltrates have an odds ratio of 1.35, but its confidence interval covers 1 (0.76-2.24), which means we can't statistically infer that it increases the risk for mortality. CONCLUDING: Based on the available data, it can be said that hypotension and hypoxemia significantly increase the risk of inpatient mortality. However, even though the presence of a lung infiltrate has an odds ratio greater than 1, the wide confidence interval means that it may not necessarily increase the mortality. Among the significant variables, hypotension carries a greater risk than hypoxemia due to its higher odds ratio. RESPONSE: Therefore, the most appropriate conclusion regarding these data is (A) The risk for inpatient mortality is greater for patients with hypotension than for those without hypotension.

**AI_A:** (1) RESTATING THE QUESTION: The information provided pertains to a case-control study that explores risk factors linked with inpatient mortality among elderly patients with pneumonia acquired in the community. The study outcomes are presented as odds ratios gleaned from a multivariable logistic regression model. Some predictor variables have been defined: these include hypotension, hypoxemia, and the presence of a lung infiltrate on a chest X-ray upon admission. The odds ratio for each is accompanied by a 95% confidence interval. These odds ratios allow us to infer the correlation between each risk factor and the likelihood of inpatient death. For instance, an odds ratio above 1 suggests a greater risk of inpatient mortality associated with the risk factor, whereas an odds ratio less than 1 would imply a lower risk. The main question here poses a challenge: With all other variables controlled, what conclusion about the study data is most appropriate? (2) PLANNING: Assuming the study's methodology is solid, we can start by considering each option. Among the choices given are, (A) states that patients with hypotension posses a higher mortality risk than those without it, (B) suggests hypoxemia increases mortality risk more than hypotension, (C) proposes the presence of a lung infiltrate at admission heightens mortality risk, and (D) posits that every factor significantly influences mortality risk. Next, we generate alternate assumptions. For instance, the odds ratios, as well as their associated 95% confidence intervals, might not effectively predict the contribution of each variable to the patient's mortality risk. Nonetheless, we should treat our initial assumptions as more clinically valid because the discussion is based on risk factor analysis from a rigorous case-control study. (3) CLINICAL REASONING: From the data, the odds ratio for hypotension is 3.32, with a 95% confidence interval spanning 1.65 to 5.11, which confirms a positive relationship between hypotension and inpatient mortality. The hypoxemia odds ratio is also above 1 (2.43, with a 95% confidence interval of 1.55 to 3.32), establishing a similar link to increased risk. On the other hand, the third variable (presence of lung infiltrate) has an odds ratio of 1.35. The 95% confidence interval for this variable, though, extends from 0.76 to 2.24, thus overlapping 1. This essentially means that the presence of a lung infiltrate doesn't significantly alter the risk of mortality since the range includes the possibility of no effect (odds ratio of 1). (4) CONCLUDING: These evidence-based reasoning steps lead us to infer that while hypoxemia and hypotension noticeably impact mortality risk, a co-existing lung infiltrate might not significantly alter the odds. (5) RESPONSE: Given these analyses, the statement most accurately describing this scenario is (A) The risk for inpatient mortality is greater for patients with hypotension than for those without hypotension. This is supported by the highest odds ratio observed for hypotension. Even though hypoxemia also increases the risk, its odds ratio is lower than that of hypotension (2.43 vs 3.32). Lastly, the odds ratio for the presence of a lung infiltrate is not significantly distinct from 1, implying that it's not a strong predictor of mortality.

**Facilitator** The consensus statement synthesizing the responses of AI_A, AI_B, AI_C, AI_D, and AI_E is as follows: The data provided is from a case-control study that investigates risk factors predicting inpatient mortality among elderly patients with community-acquired pneumonia. The predictor variables analyzed include hypotension, hypoxemia, and the presence of a lung infiltrate on a chest x-ray at admission. The odds ratios for these variables were calculated using a multivariable logistic regression model, and each is accompanied by a 95% confidence interval. The odds ratios and their respective 95% confidence intervals allow us to infer the correlation between each risk factor and the likelihood of inpatient death. An odds ratio above 1 suggests a greater risk of inpatient mortality associated with the risk factor, whereas an odds ratio less than 1 would imply a lower risk. The 95% confidence interval provides a plausible range of the true odds ratios, and if it includes 1, it indicates that the predictor may not have a significant effect. Upon analyzing the data, it is observed that both hypotension and hypoxemia have odds ratios significantly above 1, and their 95% confidence intervals do not include 1, indicating a significant association with inpatient mortality. However, the odds ratio for the presence of a lung infiltrate is not significantly distinct from 1, and its confidence interval includes 1, implying that it's not a strong predictor of mortality. Therefore, the most appropriate conclusion regarding these data is (A) The risk for inpatient mortality is greater for patients with hypotension than for those without hypotension. This conclusion is supported by the highest odds ratio observed for hypotension, indicating a stronger association with mortality when compared to hypoxemia. The presence or absence of a lung infiltrate does not significantly alter the mortality risk in these patients.
